# Thin Strut CoCr Biodegradable Polymer Biolimus A9-Eluting Stents versus Thicker Strut Stainless Steel Biodegradable Polymer Biolimus A9-Eluting Stents: Two-Year Clinical Outcomes

**DOI:** 10.1155/2021/6654515

**Published:** 2021-04-01

**Authors:** Ian B. A. Menown, Mamas A. Mamas, James M. Cotton, David Hildick-Smith, Franz R. Eberli, Gregor Leibundgut, Damras Tresukosol, Carlos Macaya, Samuel Copt, Sara Sadozai Slama, Keith G. Oldroyd

**Affiliations:** ^1^Craigavon Cardiac Centre and Queens University, Belfast, UK; ^2^University Hospital of North Midlands NHS Trust, Keele University, Keele, UK; ^3^Royal Wolverhampton NHS Trust, Wolverhampton, UK; ^4^Brighton and Sussex University Hospitals, Brighton, UK; ^5^Triemli Hospital, Zurich, Switzerland; ^6^Kantonsspital Baselland, Liestal, Switzerland; ^7^Her Majesty Cardiac Center, Bangkok, Thailand; ^8^Hospital Clinico San Carlos, Madrid, Spain; ^9^Biosensors Clinical Research, Morges, Switzerland

## Abstract

**Background:**

While thinner struts are associated with improved clinical outcomes in bare-metal stents (BMS), reducing strut thickness may affect drug delivery from drug-eluting stents (DES) and there are limited data comparing otherwise similar thin and thick strut DES. We assessed 2-year outcomes of patients treated with a thin strut (84–88um) cobalt-chromium, biodegradable polymer, Biolimus A9-eluting stent (CoCr-BP-BES) and compared these to patients treated with a stainless steel, biodegradable polymer, Biolimus A9-eluting stent (SS-BP-BES).

**Methods:**

In total, 1257 patients were studied: 400 patients from 12 centres receiving ≥1 CoCr-BP-BES in the prospective Biomatrix Alpha registry underwent prespecified comparison with 857 patients who received ≥1 Biomatrix Flex SS-BP-BES in the LEADERS study (historical control). The primary outcome was major adverse cardiac events (MACE)—cardiac death, myocardial infarction (MI), or clinically driven target vessel revascularization (cd-TVR). Propensity analysis was used to adjust for differences in baseline variables and a landmark analysis at day-3 to account for differences in periprocedural MI definitions.

**Results:**

MACE at 2 years occurred in 6.65% CoCr-BP-BES versus 13.23% SS-BP-BES groups (unadjusted HR 0.48 [0.31–0.73]; *P*=0.0005). Following propensity analysis, 2-year adjusted MACE rates were 7.4% versus 13.3% (HR 0.53 [0.35–0.79]; *P*=0.004). Definite or probable stent thrombosis, adjudicated using identical criteria in both studies, occurred less frequently with CoCr-BP-BES (1.12% vs. 3.22%; adjusted HR 0.32 [0.11–0.9]; *P*=0.034). In day-3 landmark analysis, the difference in 2-year MACE was no longer significant but there was a lower patient-orientated composite endpoint (11.7% vs. 18.4%; HR 0.6 [0.43–0.83]; *P*=0.006) and a trend to lower target vessel failure (5.8% vs. 9.1%; HR 0.63 [0.4–1.00]; *P*=0.078).

**Conclusion:**

At 2-year follow-up, propensity-adjusted analysis showed the thin strut (84–88um) Biomatrix Alpha CoCr-BP-BES was associated with improved clinical outcomes compared with the thicker strut (114–120um) Biomatrix Flex SS-BP-BES.

## 1. Introduction

Thinner stent struts may improve deliverability and conformability and reduce vessel injury. A comparison of bare-metal stents with identical design apart from strut thickness reported improved clinical outcomes with thinner struts, specifically a lower incidence of angiographic restenosis and repeat revascularization [1]. However, reducing strut thickness may affect drug delivery from drug-eluting stents (DES). Furthermore, most trials evaluating DES with thinner versus thicker struts have compared stents with different designs and/or different polymers and/or different drugs [2]. We thus aimed to assess the clinical impact of reducing strut thickness in DES by comparing two otherwise similar DES, apart from a difference in strut thickness. We also assessed clinical outcomes to 2 years to mitigate the potentially confounding effect of different durations of dual antiplatelet therapy (DAPT) in earlier follow-up.

## 2. Materials and Methods

### 2.1. Stent Design

In this paper, we describe 2-year outcomes after a percutaneous coronary intervention (PCI) with the Biomatrix Alpha^TM^ cobalt-chromium, biodegradable polymer, Biolimus A9 eluting stent (CoCr-BP-BES) and the Biomatrix Flex^TM^ stainless steel, biodegradable polymer, Biolimus A9 eluting stent (SS-BP-BES). Both stents are abluminally coated with a mixture of Biolimus A9 and a polylactic acid (PLA) polymer matrix (50 : 50 by weight) in a dose of 15.6 *µ*g/mm stent length. Biolimus A9 is an m-TOR inhibitor with a cytostatic mechanism of action similar to sirolimus but custom-designed with a ligand modification which results in 10-fold increased lipophilicity. PLA is biodegradable and fully absorbed within 6–9 months. While drug and polymer are identical in formulation and dose, the Biomatrix Alpha^TM^ CoCr-BP-BES platform is made from cobalt-chromium (MP35 N) which has enabled a reduction in strut thickness from 114–120 *µ*m (in the 316L stainless steel Biomatrix Flex^TM^ SS-BP-BES) to 84–88 *µ*m while maintaining similar radial strength. All other stent design elements have remained unchanged including the hybrid design of mid-section S-connectors for improved flexibility combined with straight connectors for higher longitudinal strength in the proximal and distal end sections of the stent [3].

### 2.2. Study Design and Patients

The Biomatrix Alpha™ Registry [3] was a prospective, single-arm, multicentre registry conducted in 12 centres across 4 countries in Europe and Asia, which enrolled 400 patients with stable coronary artery disease or acute coronary syndrome receiving at least one Biomatrix Alpha^TM^ CoCr-BP-BES. Patients were eligible for inclusion if they had undergone PCI in one or more coronary arteries or bypass grafts. There were no limitations as to the number of treated vessels, or the number, type, and length of treated lesions. Patients were excluded if any additional stent(s) different from the study stent were implanted during the index procedure. The registry was managed by the Cardiovascular European Research Center (CERC) in Massy, France. The primary endpoint was the incidence of major adverse cardiac events (MACE) at 9 months—a composite of cardiac death, myocardial infarction (MI), and clinically driven target vessel revascularization (cd-TVR). We previously reported the incidence of MACE at 9 months to be 3.94% (95% CI [2.39–6.47]) which met criteria for noninferiority (*P* < 0.001) versus the SS-BP-BES arm of the LEADERS study [4]. In this paper, we report clinical outcomes up to 2 years. Secondary endpoints included target vessel failure (TVF), a composite of cardiac death or target vessel (TV), MI, or cd-TVR; the patient-oriented composite endpoint (POCE), a composite of all-cause mortality, any MI, or any revascularization; individual components of the composite endpoints; and ARC definite or probable stent thrombosis.

The LEADERS study [4] was a randomized comparison of 857 patients receiving at least one SS-BP-BES (Biomatrix Flex^TM^) versus 850 patients receiving a stainless steel, permanent polymer, and sirolimus-eluting stent (SS-PP-SES) (Cypher™, Cordis, Miami Lakes, FL, USA). The SS-BP-BES group showed noninferiority with respect to MACE at 9 months (which was maintained at 5 years) and a significant reduction in very late definite ST from 1 to 5 years compared with the SS-PP-SES group [5]. Key elements of the Biomatrix Alpha registry protocol were intentionally kept the same as in the LEADERS study, to enable a prespecified comparison of patients receiving CoCr-BP-BES stents versus patients in the SS-BP-BES arm of the LEADERS study [4] as a historic control. DAPT with aspirin and a P_2_Y_12_ inhibitor was recommended as per clinical practice guidelines in the Biomatrix Alpha registry and for at least one year in the LEADERS study.

### 2.3. Data and definitions

Both studies were conducted in accordance with good clinical practice (GCP) guidelines and the 1975 Declaration of Helsinki. Both were registered with Clinicaltrials.gov and informed consent from each patient was obtained. Baseline data have been described previously [3, 4]. All reported MACE and stent thrombosis events in both studies were monitored, checked against source documents, and adjudicated by an independent Clinical Event Committee (CEC). Cardiac death was defined as any death due to immediate cardiac cause (e.g., MI, low-output failure, fatal arrhythmia), unwitnessed death, or death of unknown cause. In the Biomatrix Alpha registry, MI was defined by the Third Universal Definition of MI [6]. In the LEADERS study, MI was defined by Minnesota code ECG criteria or creatine kinase (CK) >2x upper limit of normal with elevated CK-MB or troponin. cd-TVR was defined as a repeat PCI or bypass surgery of the target vessel associated with either *a* ≥ 70% vessel diameter reduction or *a* ≥ 50% diameter reduction in combination with angina and/or documented ischemia. Stent thrombosis was categorized as definite or probable according to the Academic Research Consortium (ARC) definitions [7, 8] with all relevant angiograms reviewed by the CEC.

### 2.4. Statistical Analysis

For continuous variables, mean and standard deviation are reported. For categorical variables, counts and percentages are shown. The denominator for the calculation of percentages is based upon the number of the nonmissing values available unless otherwise specified. Clinical events are reported as Kaplan–Meier estimates with corresponding confidence intervals based on the log-log transformation and hazard ratio (HR) derived from the Cox proportional hazard model. All data were analysed using SAS V.9.4 (SAS Institute, Cary, North Carolina, USA).

In order to adjust for potential baseline differences, we conducted a patient-level propensity score analysis [9–11] between the datasets of the CoCr-BP-BES in the Alpha registry and the SS-BP-BES arm of LEADERS. The propensity for each patient was modelled as the probability of being part of the Alpha registry versus being part of the SS-BP-BES arm in LEADERS. The propensity scores were obtained by inverse probability of treatment weight (IPTW). The full list of baseline variables used in the propensity score calculation is provided in Supplementary Table S1.

While the Biomatrix Alpha Registry protocol was designed to match the LEADERS protocol, post-PCI biomarkers were encouraged in the Alpha Registry but mandatory in the LEADERS study and the updated Third Universal Definition of myocardial infarction [6] was used only in the Alpha Registry. Recognizing that different definitions might introduce a potential discrepancy in MI reporting between the Alpha registry and the LEADERS study, particularly for periprocedural MI (within 48 hours), we then conducted a landmark analysis censoring clinical events which were part of the primary endpoint occurring up to day 3.

## 3. Results

### 3.1. Patient and Lesion Characteristics

Baseline patient and lesion characteristics of CoCr-BP-BES versus SS-BP-BES are shown in Table 1. The mean age in the two groups was CoCr-BP-BES 64.7 ± 11 years versus SS-BP-BES 64.6 ± 10.8 years and 21% versus 24%, respectively, were current smokers. The proportion of diabetes patients differed between the two groups (CoCr-BP-BES 19.3% vs. SS-BP-BES 26.1%). Over half of the patients in both groups presented with an acute coronary syndrome (acute MI or unstable angina). Renal insufficiency was more common in the CoCr-BP_BES group (11.5% vs. 5.4%) but prior revascularization was more common in the LEADERS study (24.6% vs. 40.9%). Procedural details are listed in Table 1. Use of DAPT in the Alpha registry versus LEADERS study was lower at all time points (96% vs. 98%; *P* < 0.001 at 30 days, dropping to 69% vs. 93%; *P* < 0.0001 at 9 months and 0% vs. 21%; *P* < 0.0001 at 2 years) reflecting evolving treatment guidelines, different proportions of ACS patients, and greater use of single antiplatelet therapy plus oral anticoagulation.

### 3.2. Clinical Outcomes

**Figure 1:**
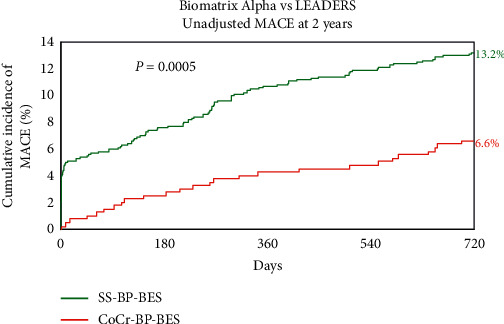
Unadjusted MACE at 2 years. CoCr-BP-BES = cobalt-chromium biodegradable polymer Biolimus A9-eluting stent, MACE = major cardiac adverse events, and SS-BP-BES = stainless steel biodegradable polymer Biolimus A9-eluting stent.

Previously published 9-month MACE outcomes met criteria for noninferiority [3]. The unadjusted difference in MACE remained consistent at 2 years (6.6% with CoCr-BP-BES vs. 13.2% with SS-BP-BES; HR 0.48[0.31–0.73]; *P*=0.0005) (Figure 1). Individual components of the composite MACE endpoint and unadjusted secondary endpoints are listed in Table 2. Each component of MACE and most secondary endpoints were significantly lower in the CoCr-BP-SES group. A Cox regression multivariate analysis, including 15 baseline characteristics (Table S1) and stent type, found the stent type to be an independent predictor of MACE (*P*=0.0028).

### 3.3. Propensity Analysis

**Figure 2:**
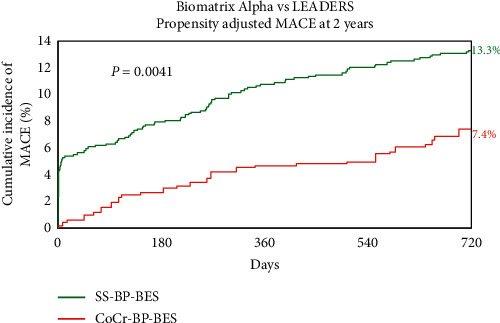
Propensity-adjusted MACE at 2 years. CoCr-BP-BES = cobalt-chromium biodegradable polymer Biolimus A9-eluting stent, MACE = major cardiac adverse events, and SS-BP-BES = stainless steel biodegradable polymer Biolimus A9-eluting stent.

Given differences in patient baseline characteristics despite using matching inclusion criteria, a propensity analysis was undertaken and adjusted for 15 variables.  Figure 2 shows that the difference in MACE remained after propensity adjustment (7.4% vs. 13.3%; HR 0.52 [0.35 : 0.79]; *P*=0.0041). Similar to the unadjusted data, the difference in MACE emerged early then remained consistent up to 2 years. Supplementary Figure 1 shows the incidence of MACE during the first month versus months 2–24.

### 3.4. Stent Thrombosis

**Figure 3:**
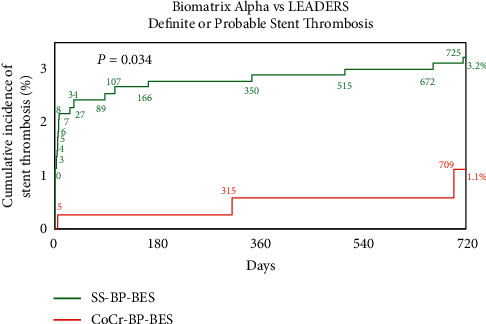
Propensity-adjusted definite or probable stent thrombosis at 2 years. Stent thrombosis was adjudicated using identical criteria in both studies. CoCr-BP-BES = cobalt-chromium biodegradable polymer Biolimus A9-eluting stent, MACE = major cardiac adverse events, and SS-BP-BES = stainless steel biodegradable polymer Biolimus A9-eluting stent.

The safety endpoint of definite or probable stent thrombosis was adjudicated using identical ARC criteria in both studies.  Figure 3 shows that after propensity adjustment, the incidence of definite or probable stent thrombosis was markedly lower at 2 years with CoCr-BP-DES (1.1 vs. 3.2%; HR 0.32 [0.114 : 0.897]; *P*=0.034) with most of the difference being in early stent thrombosis.

Of note, the reduction of definite or probable stent thrombosis with CoCr-BP-DES was achieved despite a shorter duration of DAPT.

### 3.5. Landmark Analysis at Day 3

**Figure 4:**
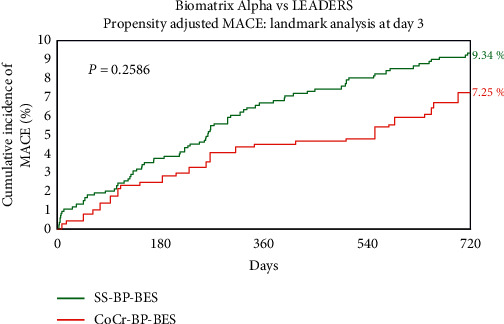
Propensity-adjusted MACE at 2 years with a landmark at day 3. CoCr-BP-BES = cobalt-chromium biodegradable polymer Biolimus A9-eluting stent, MACE = major cardiac adverse events, SS-BP-BES = stainless steel biodegradable polymer Biolimus A9-eluting stent.

To account for possible differences in MI reporting, particularly for periprocedural (Type 4a) MI, we conducted a landmark analysis censoring clinical events contributing to the primary endpoint that occurred up to and including day 3. Following landmark analysis, the MACE rate with CoCr-BP-DES remained numerically lower than with SS-BP-BES (Figure 4) but was no longer statistically significant (7.25% vs. 9.34%; HR 0.76 [0.5–1.17]; *P*=0.25). Individual elements of the composite MACE endpoint and adjusted secondary endpoints following landmark analysis at day 3 are shown in Table 3. The patient-oriented composite endpoint remained significantly lower with CoCr-BP-BES (11.7% vs. 18.7%; HR 0.6[0.43–0.83]; *P*=0.006) and there was a trend to less frequent target vessel revascularization (5.8% vs. 9.1%; HR 0.63 [0.4–1.00]; *P*=0.078).

## 4. Discussion

In this first report of longer-term (2-year) outcomes of patients undergoing PCI with CoCr-BP-BES and at a time point when all patients had discontinued dual antiplatelet therapy, the unadjusted event rates with CoCr-BP-BES remained low. These results are in keeping with recent data from studies using other 3^rd^ generation DES. The BIO-RESORT randomized trial of 3514 all-comer patients [12] and reported 2-year TVF rates of 6.8% for the Synergy^TM^ biodegradable polymer everolimus-eluting stent, 6.6% for the Orsiro^TM^ CoCr-BP-SES, and 8.3% for the RESOLUTE^TM^ permanent polymer zotarolimus-eluting stent (ZES) (*P*=ns). In the BIONYX trial [13], 2-year TVF rates were 7.6% with the Resolute Onyx^TM^ permanent polymer ZES and 7.1% with Orsiro^TM^

While thinner stent struts are associated with factors such as reduced wall shear stress [14] and less malapposition [15], both of which may reduce thrombotic risk, thinner struts are more prone to longitudinal compression [16] and to an increased risk of tissue prolapse increasing the thrombotic risk [17]. Thinner struts may also impact on strut spacing and impact local drug diffusion. It is thus important to study DES strut thickness directly rather than extrapolate from BMS data.

Previous DES studies have compared dissimilar technologies with differences in stent design, polymer, and drug [2] which may confound analysis. While a meta-analysis of thinner strut CoCr versus thicker strut SS-DES showed a reduction in MI at 30 days [18], in SORT OUT VII, the Orsiro^TM^ CoCr-BP-SES failed to show a significant reduction in target lesion failure at 3 years compared with the Nobori^TM^ SS-BP-BES despite a marked difference in strut thickness (60–80 *µ*m vs. 114–120 *µ*m) [19]. While our comparison was not randomized, the prespecified protocol of the Biomatrix Alpha Registry facilitated the comparison of thin (84–88 *µ*m) versus thicker (114–120 *µ*m) struts in the 3^rd^ generation stents while controlling for the stent design, polymer, and drug.

Although MI definitions differed between the 2 studies, definite or probable stent thrombosis was adjudicated using identical criteria. It was thus appropriate to report its incidence without landmark adjustment. The reduction in definite or probable stent thrombosis rates is notable and is consistent with previous literature [2] suggesting that this may be the principal benefit of reduced strut thickness.

The strong trend towards lower cd-TVR with Biomatrix Alpha shows that the antiproliferative effect of Biolimus was not compromised despite the thinner strut platform.

The limitations of this study are the modest sample size and the use of historical rather than prospectively randomized controls. It is possible that some of the outcome benefits described are related to advances in procedural techniques and concomitant drug therapy over the past decade, not fully adjusted for in the propensity analysis. In line with typical registry protocols, only 10% of the patients in the Alpha registry were fully monitored, thus there is a possibility of underreporting of clinical events, although 100% adjudication of MACE events was undertaken.

## 5. Conclusion

In this analysis, 2-year clinical outcomes with the thin strut (84–88um) Biomatrix Alpha^TM^ CoCr-BP-BES were excellent with rates of MACE at 6.6%, TVF 5.1%, and definite/probable stent thrombosis 0.8%. A prespecified propensity-adjusted analysis showed improved clinical outcomes compared with the thicker strut (114–120 *µ*m) Biomatrix Flex^TM^ SS-BP-BES.

## Figures and Tables

**Table 1:** Baseline demographics and procedural details.

	CoCr-BP-BES *n* = 400	SS-BP-BES *n* = 857	*P* value
*Baseline demographics*			
** **Mean age (years)	64.7 ± 11	64.6 ± 10.8	0.892
** **Female gender (%)	21.5	25	0.178
** **STEMI or NSTEMI (%)	41.1	32.7	0.004
** **Unstable angina (%)	14	22.2	<0.001
** **Prior MI (%)	18.8	32.2	<0.0001
** **Previous PCI or CABG (%)	24.6	40.9	<0.0001
** **Previous stroke (%)	6.3	4.7	0.292
** **Current smoker (%)	21	24.1	0.229
** **Hypertension (%)	57.3	73.6	<0.0001
** **Dyslipidemia (%)	56.7	65.4	0.003
** **Diabetes (%)	19.3	26.1	0.009
** **Renal insufficiency (%)	11.5	5.4	<0.0001

*Procedural details*			
** **Staged procedure (%)	5.5	4.4	0.476
** **Target lesion coronary artery (%)			
** **LAD	47.4	37.2	<0.0001
** **LCX	20.1	28	<0.001
** **LM	2.3	2.6	0.399
** **RCA	26.9	30.7	0.112
** **De novo lesions (%)	95.9	94	0.123
** **Bifurcation lesions (%)	25.8	22.4	0.132
** **Number of stents per lesion	1.34 ± 0.70	1.20 ± 0.48	<0.0001
** **Severe calcification (%)	16.2	13.1	0.09
** **Lesion length (mm)	21.7 ± 12.8	15.2 ± 11.7	<0.0001
** **Reference vessel diameter (mm)	3.0 ± 0.5	2.6 ± 0.61	<0.0001

CABG=coronary artery bypass grafting, CoCr-BP-BES = cobalt-chromium biodegradable polymer Biolimus A9-eluting stent, MI = myocardial infarction, PCI = percutaneous coronary intervention, and SS-BP-BES = stainless steel biodegradable polymer Biolimus A9-eluting stent.

**Table 2:** Biomatrix Alpha versus LEADERS : unadjusted MACE at 2 years.

	CoCr-BP-BES (*n* = 400)	SS-BP-BES (*n* = 857)	Hazard ratio	*P* value
MACE	26 (6.65%)	112 (13.23%)	0.48 [0.31–0.73]	0.0005
- Cardiac death	4 (1.01%)	27 (3.21%)	0.31 [0.11–0.89]	0.022
- MI	12 (3.13%)	55 (6.48%)	0.46 [0.24–0.85]	0.012
- cd-TVR	16 (4.09%)	65 (7.8%)	0.51 [0.3–0.89]	0.0152
All death	15 (3.82%)	40 (4.72%)	0.79 [0.44–1.44]	0.449
Target vessel MI	5 (1.29%)	27 (3.18%)	0.39 [0.15–1.03]	0.048
Definite or probable stent thrombosis	3 (0.81%)	26 (3.07%)	0.25 [0.08–0.82]	0.013
Any revasc	29 (7.46%)	143 (17.14%)	0.40 [0.27–0.60]	<0.0001
TVF (cardiac death or TV-MI or cd-TVR)	20 (5.09%)	96 (11.36%)	0.43 [0.27–0.7]	0.0004
POCE (all death or any MI or any revasc)	43 (10.9%)	192 (22.58%)	0.44 [0.32–0.61]	<0.0001

revasc = revascularization, cd-TVR = clinically driven target vessel revascularization, CoCr-BP-BES = cobalt-chromium biodegradable polymer Biolimus A9-eluting stent, MACE = major adverse cardiac events, MI = myocardial infarction, POCE = patient-oriented composite endpoint, SS-BP-BES = stainless steel biodegradable polymer Biolimus A9-eluting stent, and TVF = target vessel failure.

**Table 3:** Biomatrix Alpha versus LEADERS : propensity-adjusted MACE at 2 years with a landmark at day 3.

	CoCr-BP-BES (%)	SS-BP-BES (%)	Hazard ratio	*P* value
MACE	7.25	9.34	0.76 [0.5–1.17]	0.259
- Cardiac death	1.29	3.26	0.39 [0.15–1.00]	0.064
- Myocardial infarction	2.82	2.36	1.17 [0.56–2.47]	0.721
- cd-TVR	4.57	6.39	0.65 [0.38–1.10]	0.152
All death	4.12	4.74	0.86 [0.49–1.52]	0.638
Target vessel MI	0.90	0.91	1.01 [0.28–3.59]	0.991
Definite or probable stent thrombosis	1.12	2.11	0.5 [0.17–1.45]	0.238
Any revasc	8.20	15.47	0.5 [0.34–0.73]	0.001
TVF (cardiac death or TV-MI or cd-TVR)	5.83	9.08	0.63 [0.4–1.00]	0.078
POCE (all death or any MI or any revasc)	11.69	18.39	0.6 [0.43–0.83]	0.006

## Data Availability

The data used for the study endpoints may be made available on request from the corresponding author.

## Supplementary Materials

Supplementary Materials The full list of baseline variables used in the propensity score calculation and weighted *p* values is provided in Supplementary Table S1. Supplementary Figure S1 shows the incidence of MACE (propensity-adjusted) with landmark analysis at 30 days.
