# Supplementary material for: Thin Strut CoCr Biodegradable Polymer Biolimus A9-Eluting Stents versus Thicker Strut Stainless Steel Biodegradable Polymer Biolimus A9-Eluting Stents: Two-Year Clinical Outcomes
Source: J Interv Cardiol. 2021 Apr 1;2021:6654515. doi: 10.1155/2021/6654515 (PMC8032541; doi:10.1155/2021/6654515)
Supplement: Supplementary Materials — The full list of baseline variables used in the propensity score calculation and weighted p values is provided in Supplementary Table S1. Supplementary Figure S1 shows the incidence of MACE (propensity-adjusted) with landmark analysis at 30 days. [file 6654515.f1.zip › 6654515.f1/Supplementary table S1_Menown (1).docx]

|  | |  |
| --- | --- | --- |
|  | P values |  |
| Mean age (years) | 0.689 |  |
| Female gender (%) | 0.523 |  |
| Body mass index | 0.426 |  |
| Family History | 0.707 |  |
| Current smoker (%) | 0.541 |  |
| Hypertension (%) | 0.505 |  |
| Dyslipidemia (%) | 0.585 |  |
| Diabetes (%) | 0.614 |  |
| Renal insufficiency (%) | 0.934 |  |
| Previous Stroke | 0.971 |  |
| Prior MI (%) | 0.953 |  |
| Previous PCI or CABG | 0.508 |  |
| STEMI (%) | 0.894 |  |
| Staged procedure | 0.9 |  |
| Multi-lesion procedure (%) | 0.881 |  |

*Table S1: Weighted p values (after IPTW adjustment) CoCr-BP-BES vs. SS-BP-BES*

CABG=Coronary artery bypass grafting

MI=myocardial infarction

PCI=percutaneous coronary intervention
