# Supplementary figures and images for: Thin Strut CoCr Biodegradable Polymer Biolimus A9-Eluting Stents versus Thicker Strut Stainless Steel Biodegradable Polymer Biolimus A9-Eluting Stents: Two-Year Clinical Outcomes
Source: J Interv Cardiol. 2021 Apr 1;2021:6654515. doi: 10.1155/2021/6654515 (PMC8032541; doi:10.1155/2021/6654515)

## Slide 1
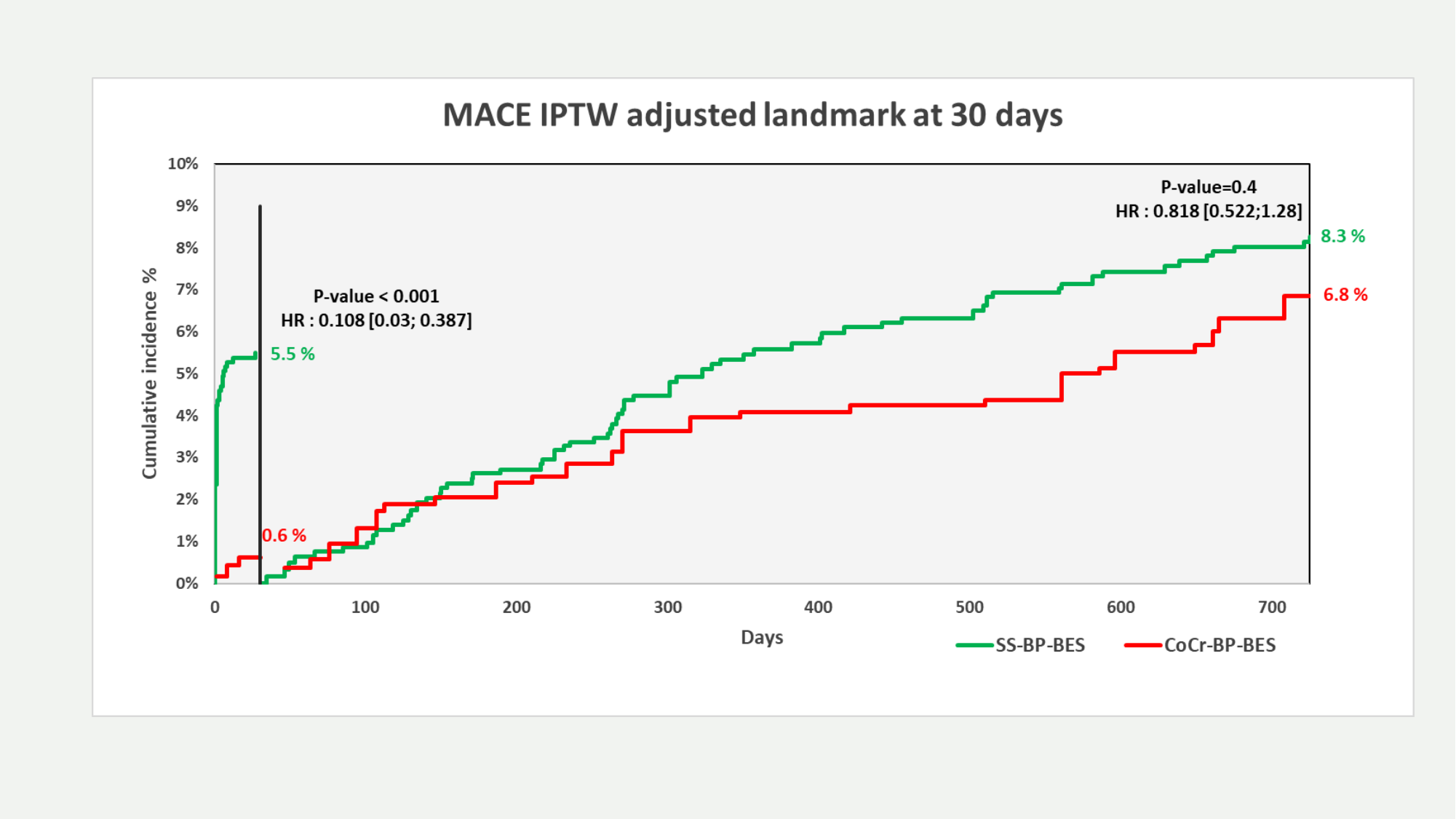

Supplement: Supplementary Materials — The full list of baseline variables used in the propensity score calculation and weighted p values is provided in Supplementary Table S1. Supplementary Figure S1 shows the incidence of MACE (propensity-adjusted) with landmark analysis at 30 days. [file 6654515.f1.zip › 6654515.f1/Supplementary fig S1 Menown.pptx]
